# Association of children wheezing diseases with meteorological and environmental factors in Suzhou, China

**DOI:** 10.1038/s41598-022-08985-5

**Published:** 2022-03-23

**Authors:** Jia-qi Huang, Jin Zhang, Chuang-li Hao, Zheng-rong Chen

**Affiliations:** Department of Respiratory Disease, Children’s Hospital of Soochow University, Jingde Road NO. 303, Suzhou, 215003 Jiangsu China

**Keywords:** Environmental sciences, Diseases, Medical research, Risk factors

## Abstract

Wheezing diseases are one of the major chronic respiratory diseases in children. To explore the effects of meteorological and environmental factors on the prevalence of children wheezing diseases, clinical data of children hospitalized with wheezing diseases in Suzhou, China from 2013 to 2017 were collected. Meteorological and environmental factors from 2013 to 2017 were obtained from the local Meteorological Bureau and Environmental Protection Bureau. Relationships between wheezing diseases and meteorological and environmental factors were evaluated using Pearson’s correlation and multivariate regression analysis. An autoregressive integrated moving average (ARIMA) model was used to estimate the effects of meteorological and environmental variables on children wheezing diseases. Children wheezing diseases were frequently presented in infants less than 12 months old (1897/2655, 58.28%), and the hospitalization rate was highest in winter (1024/3255, 31.46%). In pathogen-positive specimens, the top three pathogens were respiratory syncytial virus (21.35%), human rhinovirus (16.28%) and mycoplasma pneumoniae (10.47%). The seasonality of wheezing children number showed a distinctive winter peak. Children wheezing diseases were negatively correlated with average temperature (P < 0.001, r =  − 0.598). The ARIMA (1,0,0)(0,0,0)_12_ model could be used to predict temperature changes associated wheezing diseases. Meteorological and environmental factors were associated with the number of hospitalized children with wheezing diseases and can be used as early warning indicators for the occurrence of wheezing diseases and prevalence of virus.

## Introduction

Wheezing is the most common and specific symptom associated with asthma in young children^1^. It has been reported that one-third of children have had at least one wheezing episode before the age of 2 years and 50% of children have it before the age of 6 years. Moreover, 40% of children with wheezing diseases will continue to wheeze after childhood^2^, and the prevalence of repeated wheezing in infants less than 1 year is more than 10%^3–5^. The global prevalence associated with childhood asthma has increased significantly over the past 40 years, which is a crucial cause of children's medical treatment and hospitalization, affecting children’s health and causing a large economic burden on the family and society.

Children wheezing diseases include low respiratory tract infections (LRTIs) with wheezing and acute exacerbation of asthma caused by infection^6^. Wheezing is a lower respiratory tract symptom induced by various viral respiratory infections^7^. Related study shows that respiratory virus infection is the main cause of wheezing in children, approximately 80% of children with acute wheezing had respiratory viral infection^8^. It mainly includes respiratory syncytial virus (RSV), human rhinovirus (HRV), adenovirus (ADV), influenza virus types A and B (Inf-A and Inf-B), and parainfluenza virus I/II/III (Pinf I–III), human metapneumovirus (hMPV), and human bocavirus (HBoV)^7,9–11^. Recurrent infections of the lower respiratory tract can present as recurrent wheezing^12^, causing LRTIs with wheezing including bronchiolitis, bronchitis with wheezing, pneumonia with wheezing, and acute exacerbation of asthma caused by infection^13–16^.

Previous studies have confirmed that age, climate, air pollution, obesity, and breastfeeding of children are inextricably linked with the occurrence of wheezing diseases in children^17–19^. An increasing number of studies have reported that meteorological and environmental factors were associated with wheezing diseases, and found that air pollution has become the ninth risk factor for death in human heart and lung diseases^20^.

Several epidemiological studies showed that prolonged exposure to air pollution or a terrible environment could causally contribute to the exacerbation of asthma symptoms. Pollution from traffic and industry such as PM_10_, PM_2.5_, NO, and NO_2_ could cause higher asthma prevalence after the school-age during childhood^21^. Indoor allergen exposure and environmental tobacco smoke were predisposing wheezing factors to onset and persistence^22^. Literature reported that air pollutants other than CO were positively associated with hospitalizations for asthma and acute upper and lower respiratory infection^23,24^. A study in Brazil showed that CO and O_3_ but not PM_10_ and SO_2_ were observed to be associated with hospitalizations^25^. A multicenter study summarized that PM_2.5_ played a crucial role in wheezing diseases in preschool children^26^. In addition, pneumonia in children associated with air pollutants, including PM_10_, PM_2.5_, SO_2_, O_3_, NO_2_, and CO, was well analyzed in a meta-analysis^27^. However, the meteorological and environmental factors vary from different countries and regions. Although China has conducted air pollution-related health research since the 1990s, it is limited to a few large cities and the health effects assessed are relatively one-sided. Therefore, it is necessary to provide a theoretical basis for the relationship between the occurrence and development of children wheezing diseases and meteorological and environmental factors, particularly in a typical subtropical area such as Suzhou.

In this study, we retrospectively analyzed the clinical and etiological characteristics of wheezing children in Suzhou from 2013 to 2017. The patients included were LRTIs with wheezing and acute exacerbation of asthma caused by infection. Several studies have reported that epidemic trends of common respiratory viruses were associated with meteorological and environmental factors^28–30^. Our previous study has found that meteorological factors could affect seasonality of certain respiratory virus^31^. Thus, we evaluated the association between children’s wheezing diseases and meteorological and environmental factors. The autoregressive integrated moving average (ARIMA) model was used to investigate the effects of meteorological and environmental factors on children wheezing diseases. The purpose of this study was to evaluate the effects of meteorological and environmental factors on children wheezing diseases, which may be useful for predicting the pattern of children wheezing diseases and the prevalence of virus.

## Methods

### Climate and geography of Suzhou City

Suzhou City is located in the southeast of the Yangtze River Delta (120°E, 31°N) and is a special economic zone belonging to the northern subtropical monsoon maritime climate zone. It has a warm, humid and rainy climate, with the obvious monsoon, distinct seasons, long winter and summer, and short spring and autumn. Its annual average temperature is 15–17 °C, and annual average precipitation is nearly 1076.2 mm. The population of Suzhou City grew from 6.539 million in 2013 to 10.684 million in 2017.

### Patients

The clinical data of admission patients diagnosed with LRTIs with wheezing, including bronchiolitis, bronchitis with wheezing, pneumonia with wheezing, and acute exacerbation of asthma caused by infection in the respiratory inpatient department at Children’s Hospital of Soochow University, were identified and reviewed from January 2013 to December 2017 in Suzhou. Basic information, including the child’s name, gender, age, admission date, chief complaint, radiological tests, laboratory tests diagnosis, allergic sensitization, and therapy, was collected, and a retrospective analysis was performed. This study was approved by the Institutional Human Ethical Committee of Children's Hospital of Soochow University (number: 2020CS001). All methods were performed in accordance with the relevant guidelines and regulations. Informed consent was obtained from all the guardians who participated in this study.

The wheezing diseases were defined according to the Global Initiative for Asthma^1^ and as per bronchiolitis^32^ and community-acquired pneumonia guidelines^33^. Exclusion criteria included primary immunodeficiency, cystic fibrosis, bronchopulmonary dysplasia, chronic cardio-lung diseases, bronchiectasis, tuberculosis, and active tobacco consumption as exclusion criteria.

### Meteorological and environmental data collection

Meteorological data for Suzhou, including monthly average temperature (°C), monthly average humidity (%), total monthly rainfall (mm), total monthly sunshine (h), and monthly average wind speed (m/s), were provided by the Suzhou Meteorological Bureau located at 120°E, 31°N. Environmental data, including PM_2.5_ (µg/m^3^), PM_10_ (µg/m^3^), NO_2_ (µg/m^3^), SO_2_ (µg/m^3^), CO (mg/m^3^), and O_3_ (µg/m^3^), were provided by the Suzhou Environmental Protection Bureau at 120°E, 31°N.

### Nasopharyngeal aspirates collection and detection

Samples of nasopharyngeal aspirates from children hospitalized with wheezing diseases were obtained according to a standard protocol. These samples were obtained from each patient within 24 h of admission by introducing a sterile plastic catheter into the lower pharynx via the nasal cavity. Furthermore, 3–5 mL of nasopharyngeal secretion was taken and divided into two parts after full shaking. One part of nasopharyngeal secretion was analyzed for seven common respiratory viruses, including RSV, ADV, Inf-A, Inf-B, and Pinf I–III, by using commercial slide-based assays with virus-specific fluorescence-labeled monoclonal antibodies (Light Diagnostics Respiratory Viral Screen DFA, Chemicon International, USA, from 2001 to 2005 and D3 UltraTM DFA Respiratory Virus Screening & ID Kit, Diagnostic Hybrids Inc., USA, from 2006 to 2011). HRV, hMPV and HBoV were detected using polymerase chain reaction, according to a standard protocol described previously^31^.

### Statistical analysis

The measurement data were expressed as mean ± standard deviation (${\overline{\text{x}}}$ ± s), all of which were tested for linear trend, normal distribution and homogeneity of variance. One-way analysis of variance was used to analyze the comparison between multiple groups. Associations between the number of children wheezing diseases and meteorological and environmental factors were evaluated using Pearson’s correlation analysis. Moreover, because of collinearity among meteorological and environmental factors, the associations were analyzed using multiple regression analysis; the standardized coefficients eliminate the influence of the dependent variable and the unit of the independent variable, and its absolute value directly reflects the degree of the independent variable’s influence on the dependent variable. Before the multiple linear regression analysis, the collinearity between the independent variables was diagnosed. As multicollinearity is present when the variance inflation factor (VIF) is higher than 5^34^, we used VIF of the independent variables to determine if a multicollinearity problem exists among the independent variables. Independent variables with VIF values less than 5 could be included in multiple regression. After selecting the variables, we used stepwise regression to analyze the associations between meteorological and environmental factors and the number of wheezing diseases.

At present, the ARIMA model is the most commonly used model in time series analysis, which can be used to predict diverse diseases and analyze multiple relationships between independent variables and diseases^35^. The ARIMA model contains three parameters: P, D and Q. It is displayed in the classical notation form of (p, q, d) (P, Q, D)_m_. The parameters p and P represents the lag number of time series data used in the prediction model (autoregressive lags), q and Q represent the lag number of prediction error adopted in the prediction model (moving average lags), d and D represent orders of differencing, and m indicates the cycle of month. ARIMA is expressed in mathematical form as: y_t_ = µ + ϕ_1_ * y_t-1_ + ⋯ + ϕ_p_ * y_t-p_ + θ_1_ * e_t-1_ + ⋯ + θ_q_ * e_t-q_, where y_t_ is the predicted number of wheezing children at time t, ϕ is the coefficient of AR and θ is the coefficient of MA. From the investigation of the correlation between meteorological and environmental factors and the number of wheezing children, we hope to study the predictive effect of meteorological and environmental factors on the number of wheezing children. In this case, multiple time series analysis based on ARIMA models was performed using the data from 2013 to 2016 (estimation period) to analyze the effect of meteorological and environmental factors on wheezing diseases of children. We then predicted the incidence of wheezing diseases in 2017(evaluation period) to evaluate the predictive effect of the model. Dependent variables (the number of wheezing children) were modeled as ARIMA processes with continuous predictors using IBM Statistical Package for the Social Sciences (SPSS) Expert Modeler (automatic model selection) and custom ARIMA models; this could automatically select the most suitable ARIMA model for studying the influence of meteorological and environmental factors on wheezing diseases of children. An optimal ARIMA model would be mainly diagnosed by normalized Bayesian information criterion (BIC) value, determination coefficient (R^2^), Root Mean Square Error (RMSE) and Mean Absolute Percentage Error (MAPE). The Ljung-Box Q test was used to test whether the residual series was white noise. To examine the temporal association of temperature with asthmatic diseases, we fitted the models with different lag structures from the current month (lag 0) to ≤ 2 lag months (lag 2) using the distributed lag model by SPSS Expert Modeler (automatic model selection) and custom ARIMA models. The parameters such as Estimate, Standard Error (S.E.), P-value, stationary R^2^ value, and normalized BIC were automatically selected by the expert modeling procedure.

Statistical analysis was performed using SPSS version 26.0 (IBM, New York). All statistical tests were two-tailed; a p-value of < 0.05 was considered statistically significant.

### Ethics declarations

This study was approved by the Institutional Human Ethical Committee of Children’s Hospital of Soochow University (number: 2020CS001). A written consent was obtained from all the guardians who participated in this study.

## Results

### Patient characteristics

The demographic characteristics of the children included in this study are shown in Table 1. There were 2286 males (70.23%) and 969 females (29.77%) with male/female sex ratio of 2.36:1. The age of 3255 children with wheezing disease ranged from 1 month to 14 years, with an average age of 2.17 ± 1.85 years. Children of 0–12 months old had the highest proportion of wheezing diseases. Among 3255 children with the wheezing disease, most cases were pneumonia with wheezing (83.2%, 2708/3255). Moreover, the wheezing children number varied by season (Supplementary Table S1), the highest rate of wheezing diseases was reported in winter (31.46%) and the lowest in summer (17.79%).

**Table 1:** Demographic characteristics of children.

Demographic characteristics	
Total wheezing children	3255
**Sex**	
Male	2286 (70.23%)
Female	969 (29.77%)
Age (years) (mean ± SD)	2.17 ± 1.85
**Age distribution(months)**	
0–12	1897 (58.28%)
13–36	945 (29.03%)
37–60	298 (9.16%)
> 60	115 (3.53%)
**Clinical signs**	
Wheezing	3226 (99.10%)
Cough	3189 (97.97%)
Fever	1171 (35.97%)
Running nose	846 (25.99%)
Breathlessness	178 (5.57%)
**Radiological tests**	
Patchy shadows	2139 (74.20%)
Increased lung marking	405 (14.04%)
Emphysema	21 (0.73%)
Atelectasis	13 (0.45%)
**Diagnosis**	
Bronchiolitis	53 (1.63%)
Bronchitis with wheezing	160 (6.39%)
Acute exacerbation of asthma caused by infection	334 (10.26%)
Pneumonia with wheezing	2708 (83.2%)
**Season**	
Spring	826 (25.37%)
Summer	579 (17.79%)
Autumn	826 (25.37%)
Winter	1024 (31.46%)

*SD* standard deviation.

Clinically, wheezing children mainly manifested wheezing, cough, fever, and lung rales, with an average white blood cell count of 10.72 ± 5.04 × 10^9^ cells/mL and average C-reactive protein of 7.14 ± 16.58 mg/mL. Some children had shortness of breath and dyspnea. Among the 3255 patients selected, 2079 were examined for pulmonary function tests. By 5 years of age, several children were capable of performing reproducible spirometry if coached by an experienced technician. The pulmonary function of wheezing children younger than 5 years old was tested using the tidal breathing analysis. An ECO Medics V’max 26 Pediatric pulmonary instrument (ECO Medics AG, Switzerland) was used to obtain TBFVLs and flow-time curves. 86.39% of examined patients showed obstructive ventilation dysfunction of the lower respiratory tract confirmed by tidal breathing function test, of which 565 cases were mild (27.18%), 581 cases were moderate (27.95%), and 650 cases were severe (31.27%). We performed radiological tests on 2884 children; patchy shadows were most observed among these children (Table 1). A total of 1948 patients were examined for allergic sensitization tests, showing that 21.87% of children were allergic to food and 10.27% to dust mites. For therapy, inhaled drugs are the mainstay of treatment for these wheezing children. After symptomatic treatment, these children had a good prognosis.

### Pathogen characteristics

**Figure 1:**
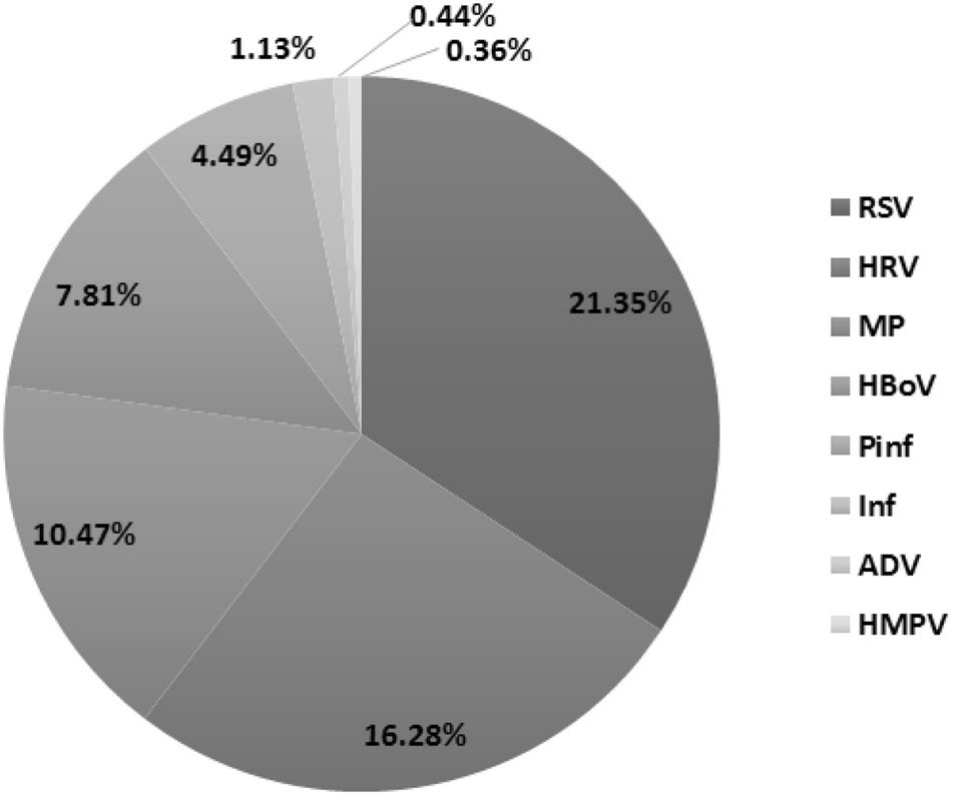
Distribution of pathogens in children with wheezing disease. RSV, respiratory syncytial virus; HRV, human rhinovirus; MP, mycoplasma pneumoniae; HBoV, human bocavirus; Pinf, parainfluenza virus; Inf, influenza virus; ADV, adenovirus; HMPV, human metapneumovirus. Figures were generated using Adobe Illustrator version CC 2018 (https://www.adobe.com/cn/products/illustrator.html).

Of the 3255 children selected, 2740 were tested for nasopharyngeal secretions, among which 795 (29.01%) were detected with one or more pathogens. The top three pathogens were RSV (21.35%), HRV (16.28%), and MP (10.47%) (Fig. 1).

### Description of meteorological and environmental data

**Figure 2:**
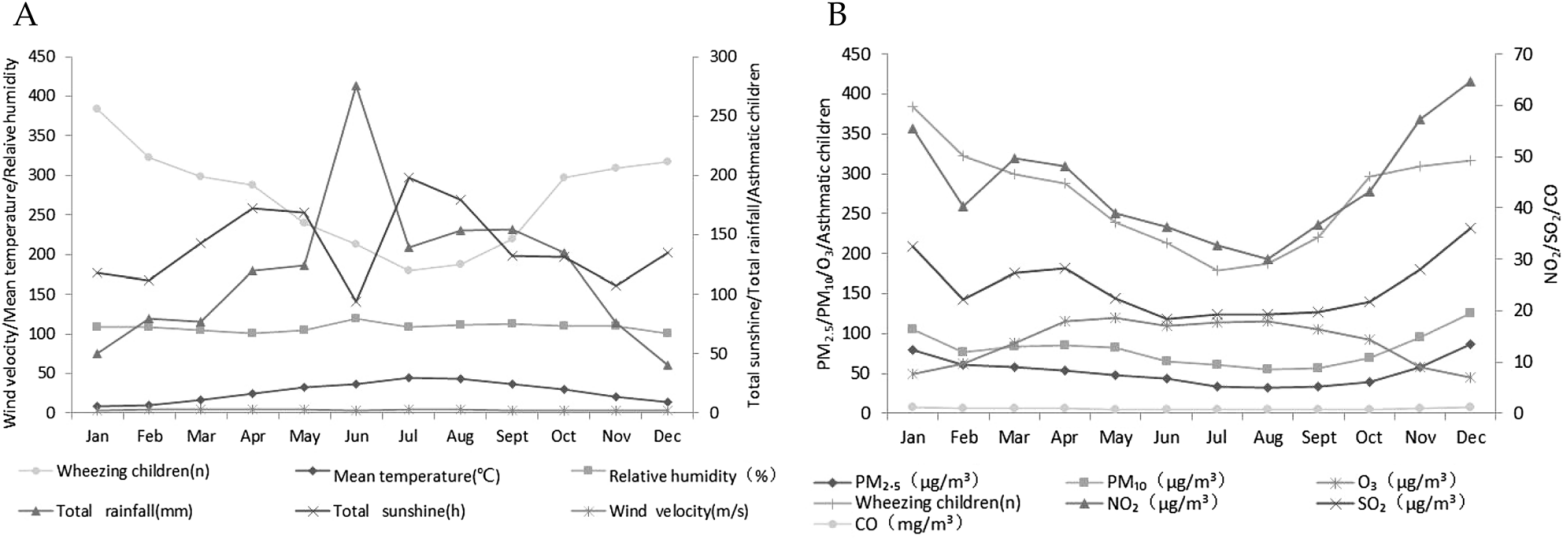
Monthly distribution of meteorological (**A**) and environmental (**B**) factors among children hospitalized with wheezing diseases. (**A**) Mean temperature, relative humidity, total rainfall, total sunshine, wind velocity and wheezing children number from 2013 to 2017 in Suzhou. (**B**) PM_2.5_, PM_10_, O_3_, NO_2_, SO_2_, CO and wheezing children number from 2013 to 2017 in Suzhou. Figures were generated using Adobe Illustrator version CC 2018 (https://www.adobe.com/cn/products/illustrator.html).

The monthly average concentrations of meteorological and environmental factors in Suzhou from 2013 to 2017 are shown in Fig. 2. The monthly average temperature was 17.7 ± 8.4 °C, average humidity was 72.3 ± 6.2%, total rainfall was 118.7 ± 90.7 mm, total sunshine was 140.9 ± 51.4 h, and the wind speed was 2.61 ± 0.37 m/s (Fig. 2A). The average monthly concentration of PM_2.5_, PM_10_, NO_2_, SO_2_ and CO showed a similar pattern with the number of wheezing children, which showed a larger peak in December (Fig. 2B). All meteorological factors were lower in winter, and temperature, humidity, and rainfall were higher in summer (Table 2). PM_2.5_, PM_10_, SO_2_, NO_2_, and CO were all higher in winter and lower in summer. However, the change of O_3_ concentration was larger in summer (Table 3).

**Table 2:** Seasonal average concentrations of meteorological factors in Suzhou area from 2013 to 2017 (${\overline{\text{x}}}$ ± s).

Season	Mean temperature (°C)	Relative humidity (%)	Total rainfall (mm)	Total sunshine (h)	Wind velocity (m/s)
Spring	16.6 ± 4.5	68.9 ± 4.6	107.0 ± 43.5	161.3 ± 35.1	2.9 ± 0.2
Summer	27.8 ± 3.0	75.3 ± 6.1	189.4 ± 116.2	157.3 ± 72.4	2.7 ± 0.2
Autumn	19.2 ± 4.7	74.3 ± 5.9	121.7 ± 91.0	123.5 ± 46.2	2.4 ± 0.3
Winter	7.0 ± 2.9	71.2 ± 6.4	59.8 ± 36.6	120.3 ± 33.5	2.4 ± 0.5
F value	69.755	3.871	6.599	2.813	8.559
P value	< 0.001	0.014	0.001	0.048	< 0.001

**Table 3:** Seasonal average concentrations of environmental factors in Suzhou area from 2013 to 2017 (${\overline{\text{x}}}$ ± s).

Season	PM_2.5_ (µg/m^3^)	PM_10_ (µg/m^3^)	NO_2_ (µg/m^3^)	SO_2_ (µg/m^3^)	O_3_ (µg/m^3^)	CO (µg/m^3^)
Spring	53.7 ± 4.6	83.6 ± 1.0	45.6 ± 4.7	26.0 ± 2.6	108.3 ± 14.0	0.8 ± 0.04
Summer	36.4 ± 5.2	60.4 ± 4.3	32.9 ± 2.5	19.0 ± 0.4	113.0 ± 2.5	0.75 ± 0.01
Autumn	44.1 ± 10.4	73.9 ± 15.8	45.8 ± 8.6	23.2 ± 3.5	85.4 ± 19.6	0.84 ± 0.11
Winter	75.8 ± 11.0	102.7 ± 19.8	53.5 ± 10.1	30.2 ± 5.8	52.6 ± 7.2	1.1 ± 0.12
F value	19.211	13.622	11.578	3.854	28.536	12.801
P value	< 0.001	< 0.001	< 0.001	0.012	< 0.001	< 0.001

### Bivariate relationship of meteorological and environmental factors with wheezing diseases

**Figure 3:**
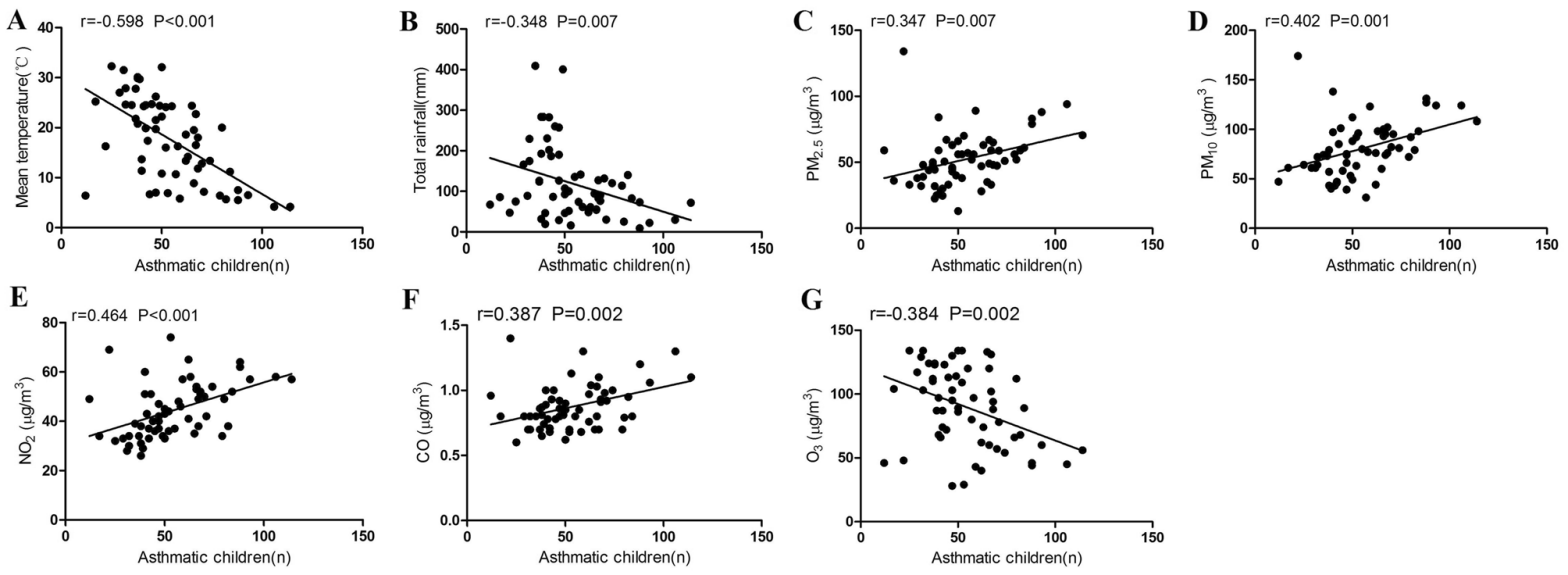
Correlation between meteorological, environmental factors and wheezing diseases in children (Pearson correlation analysis) (**A**) mean temperature; (**B**) total rainfall; (**C**) PM_2.5_; (**D**) PM_10_; (**E**) NO_2_; (**F**) CO; (**G**) O_3_. Figures were generated using GraphPad Prism version 5 (https://www.graphpad.com/scientific-software/prism/) and Adobe Illustrator version CC 2018 (https://www.adobe.com/cn/products/illustrator.html).

Using Pearson’s correlation analysis, a significant negative correlation was found between the average monthly temperature and wheezing diseases in hospitalized children (P < 0.001, r =  − 0.598) and the total monthly rainfall was also found to be negatively associated with wheezing diseases (P = 0.007, r =  − 0.348). In terms of environmental factors, PM_2.5_ (P = 0.007, r = 0.347), PM_10_ (P = 0.001, r = 0.402), NO_2_ (P < 0.001, r = 0.464), and CO (P = 0.002, r = 0.387) were positively correlated with the number of wheezing diseases, whereas O_3_ was negatively associated with the number of wheezing diseases (P = 0.002, r =  − 0.384), as shown in Fig. 3.

### Multiple regression analysis of the association between meteorological and environmental factors and wheezing diseases

Considering the correlation between various meteorological and environmental factors, multiple regression analysis was used to identify the most crucial meteorological and environmental factors. On the basis of VIF, we excluded PM_2.5_ and PM_10_ and continued to perform stepwise regression of the remaining variables. It showed a negative correlation between wheezing diseases and the monthly average temperature (Table 4). Table 5 shows no statistical difference between environmental factors and wheezing diseases. Moreover, no statistical difference was observed between sex and age range in the multiple regression of meteorological and environmental factors. Consistently, there was a negative correlation between seasonal wheezing diseases and the seasonal average temperature (Supplementary Table S2).

**Table 4:** Correlation between meteorological factors and wheezing diseases in children.

Meteorological parameter	Standardized coefficients	t	Sig	VIF
Female ratio	− 0.093	− 0.845	0.402	1.121
Average monthly age (months)	− 0.065	− 0.551	0.584	1.281
Mean temperature	− 0.647	− 3.632	0.001	2.931
Relative humidity	0.345	1.738	0.088	3.646
Total rainfall	− 0.189	− 1.220	0.228	2.218
Total sunshine	0.198	0.890	0.378	4.567
Wind velocity	0.123	1.119	0.268	1.108

Dependent variable: number of wheezing diseases, t: t value, sig: significance.

VIF: variance inflation factor.

**Table 5:** Correlation between environmental factors and wheezing diseases in children.

Environmental parameter	Standardized coefficients	T	Sig	VIF
Female ratio	− 0.106	− 0.861	0.393	1.135
Average monthly age (months)	− 0.228	− 1.739	0.088	1.286
NO_2_	0.404	1.891	0.064	3.399
SO_2_	− 0.141	− 0.836	0.407	2.127
CO	0.116	0.517	0.608	3.781
O_3_	− 0.047	− 0.263	0.794	2.353

Dependent variable: number of wheezing diseases, t: t value, sig: significance.

VIF: variance inflation factor.

### Construction of autoregressive mean sliding model

**Figure 4:**
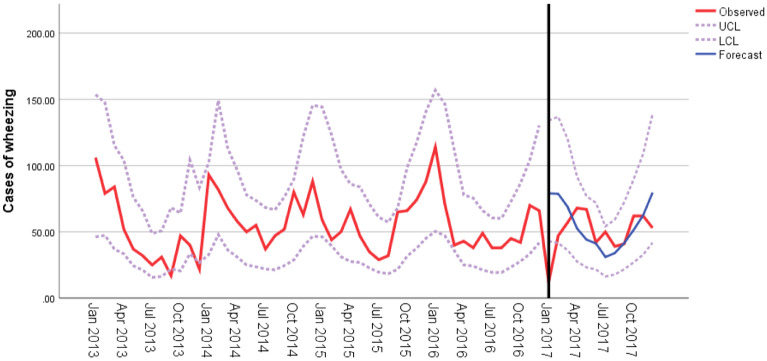
ARIMA (1,0,0)(1,1,0)_12_ model with mean temperature as the covariate. Good agreement was found between observed and predicted wheezing diseases incidence. LCL, lower confidence interval; UCL, upper confidence interval. Figures were generated using Adobe Illustrator version CC 2018 (https://www.adobe.com/cn/products/illustrator.html).

According to the above analysis results, the temperature changes were associated with the occurrence and development of wheezing diseases in children. Therefore, we established an ARIMA model on the basis of temperature changes to predict the number of wheezing diseases in hospitalized children. Figure 4 shows the results of ARIMA using an expert modeler. The ARIMA (1,0,0)(0,0,0)_12_ was the optimal model with the most suitable normalized BIC (5.575), at 15.088 of RMSE value and 25.042 of MAPE value. The stationary R^2^ value was 0.556 for the ARIMA model, which was automatically selected by the expert modeling procedure. Temperature with 0-month lag (β =  − 0.037, t =  − 5.743, P < 0.001) had a significant negative effect on the number of wheezing diseases in the ARIMA analysis. The series of residuals was white noise based on the Ljung-Box test (P = 0.599), which meets the model evaluation criteria. The actual values matched well and fell within the 95% confidence interval of the predicted values from 2013 to 2016. Figure 4 shows the predicted values of ARIMA model from January to December in 2017; each predicted value was very close to each actual value, suggesting that a predictive model for wheezing diseases in children could be established on the basis of temperature.

Consider the possibility of delayed effects that may cause some predictors omitted at this stage. The effect of temperature, rainfall, PM_2.5_, PM_10_, NO_2_, CO, O_3_ was estimated at the lag periods of 1 and 2 months using the distributed lag model. The effect of rainfall, PM_2.5_, PM_10_, NO_2_, CO, O_3_ was significant at the lag periods of 0, 1 or 2 months, whereas these independent covariants showed lower stationary R^2^ than temperature (Supplementary Table S3). Therefore, the fitting effect of temperature is the best among these independent covariants to predict the number of wheezing children.

## Discussion

Gene, environment, infection, and the body’s immune function play a crucial role in the occurrence of wheezing diseases. A close relationship has been found between changes in atmospheric pollutant concentrations and children’s wheezing diseases^36,37^. According to the World Health Organization statistics, among the deaths of children below five years old, at least one in ten child’s death is related to climate and environmental factors^38^. Therefore, it is crucial to analyze the relationship between meteorological and environmental factors and children’s respiratory diseases.

Our data demonstrated a complex correlation between children’s wheezing diseases and meteorological factors including temperature, mean relative humidity, total rain, total hours of sunshine, and mean wind velocity and environmental factors including PM_2.5_, PM_10_, NO_2_, SO_2_, CO, and O_3_. Monthly and seasonal average temperature showed more close association with wheezing diseases. The ARIMA model that incorporated temperature performed well in the prediction of wheezing diseases.

Wheezing often occurs in infants and young children whose trachea and bronchus lack elastic tissue and have a poor supporting effect. Because of poor cilia movement and clearance, their airway is typically not smooth. Moreover, they have incomplete helper T cell function, leading to scarce secretion of IgA and IgG. We included hospitalized patients with wheezing symptoms, who were mostly children below 2 years (Table 1). These truly reflected the age distribution of wheezing children. In this study, the children were LRTIs with wheezing and acute exacerbation of asthma caused by infection, and they were approximately 2 years old. Overall, these wheezing diseases were mainly caused by respiratory viral infections in infants^7^. Our previous study had reported that the prevalence of respiratory virus causing LRTIs, such as RSV, IV-A and IV-B, were affected by temperature^31^. This may influence our outcome that temperature was a crucial factor in wheezing children. However, bias may exist as outpatients were not considered. In addition, outpatients would be included in our future studies.

In this study, the temperature was identified to be the most crucial factor associated with wheezing diseases. Multiple regression analysis was used to study multicollinearity, and VIF was used to include or exclude multicollinearity at various steps. The temperature was the final factor that was associated with the wheezing disease, reflecting that temperature was a comprehensive factor that could represent other meteorological and environmental factors.

This study found that wheezing diseases peaked mainly in winter. The number of wheezing diseases in hospitalized children had a highly negative correlation with average temperature, which was consistent with the results of studies conducted in different regions^39–42^. One study conducted in Shanghai, China, found that a lower temperature was associated with a higher risk of asthma attack^40^, indicating the dangers of cold weather to wheezing children. Apart from the cold temperature, a panel study designed in six Australian cities had investigated that fluctuation in temperature also corresponded to a higher risk of an asthma attack. They found that a large intra-day temperature variation induced the risks of children’s wheezing symptoms^39^.

Children’s respiratory tracts are more sensitive to climate change; thus, a long-term temperature drop or a lower temperature level has an effect on wheezing diseases. Several pathways through which low temperature affected the occurrence of childhood asthma had been proposed in the existing literature. The low temperature had significant effects on suppressing the immune system of humans^43^, reduced the corresponding antiviral and bacterial capabilities, and increased the adrenaline secretion in the cold environment, which could also reduce the immune function. Studies have shown that an increase in temperature was associated with a decrease in lung function in children with asthma^44^. In addition, in the cold environment, the parasympathetic nervous system was stimulated to increase inflammation by generating mediators such as cysteinyl leukotrienes; thus, the contraction of the bronchial smooth muscle was increased^45^. Mechanistically, transient receptor potential melastatin 8 (TRPM8) is involved in a mechanism triggered by cold temperature. It was investigated as a mediator to trigger exacerbations of childhood asthma. TRPM8 receptor is involved in cold-induced mucus hypersecretion through the Ca(2+)-PLC-PIP2-MARCKS signaling pathway^46^. In addition, Fisher JT found that TRPM8 is a key molecule leading to respiratory sensations such as dyspnea and cold-induced asthma and cough^47^.

The statistical results of this study showed that the concentration of five pollutants had a positive correlation with the number of wheezing diseases in hospitalized children. Particulate matters, in which PM_2.5_ and PM_10_ are the most common, harbor a complex effect on wheezing airways. Several PM components are redox-active and capable of inducing cellular oxidative stress and injuries including inflammation and cell death. The convergence of regulatory signals generated by particulate matter-induced oxidative stress in dendritic cells and their interactions may also be responsible for asthma exacerbations^48^. Exposure to PM may cause acute pulmonary injury and inflammation, increasing airway responsiveness and airway remodeling, either alone or in combination with allergic sensitization^49^. Consistent with this, a study conducted by Samoli et al. found an association between exposure to PM_10_ and the occurrence of wheezing diseases^50^.

SO_2_ and NO_2_ can act as irritating gases in the respiratory tract of children. The former is known as “the culprit of atmospheric pollution” and is the main substance that induces acid rain. Several studies in recent years had found that reduced childhood pulmonary function was closely associated with NO_2_ and SO_2_ exposure^51,52^. A population-based mother–child cohort study followed 620 Spanish pregnant women, who were exposed to NO_2_ during pregnancy, from pregnancy to 4.5 years after the birth of the baby. They found that for every 10 μg/m^3^ increase in NO_2_ level, the infant’s forced expiratory volume in one second (FEV1) decreased by nearly 17.4 mL at the age of 4.5 years^51^. At the same time, the reactivity of the airway was higher with an increase in SO_2_, which led to the occurrence of wheezing^53^.

This study identified a negative association with exposure to O_3_. However, in a study conducted in São José dos Campos, a decrease in O_3_ concentrations could lead to fewer hospitalizations, although the outcome was hospitalization because of pneumonia among children^25^. Similarly, some researchers found that prolonged exposure to O_3_ could reduce lung function and increase the incidence of wheezing diseases, particularly in children^54^, which is inconsistent with our study results. This may be because of the relatively lower O_3_ concentrations in Suzhou. Furthermore, there is a close relationship between O_3_ and solar radiation. In summer, the total sunshine is longer, and the photochemical reaction is enhanced, which is conducive to O_3_ formation. In other words, the concentration of O_3_ is low in winter. The number of wheezing children rose under the influence of low temperatures, which may mask the effect of O_3_, and the associations were not obvious in this study.

There were several limitations in this study. First, this study was based on a single center in some areas of China, which may have potential biases because of the age structure and socioeconomic and production level^55^. Whether these models are suitable for other endemic areas and other infectious diseases may well require further study. We did not perform validity statistics on the ARIMA model, it is necessary to validate our conclusions with external cohorts from different countries, regions, ethnicities, and for longer periods of time. Second, in addition to meteorological and environmental factors, the factors that affected children wheezing diseases such as viral infection^56^, physical activity level^57^, and basic diseases such as rhinitis were not considered because of the availability of data. These factors might have an effect on the relationship between children’s wheezing diseases and meteorological and environmental factors. Third, the study used data at the monthly level, which in some respects did not allow for the detection of effects at the daily or weekly level, which are shorter term fluctuations. Finally, we provided limited evidence about the relationship between weather or environment and asthmatic diseases due to the limitations of statistical methods. More sophisticated models like Bayesian statistics, mathematical models and distributed lag non-linear models (DLNM) are needed to provide more evidence of environmental epidemiology and make better improvements in prediction. Despite these limitations, the source of wheezing diseases in our hospital accounted for at least 90% of the local area; therefore, we included a large number of patients to eliminate information bias, and used multiple linear regression to identify further the meteorological and environmental risk factors for wheezing children. Our study had a further understanding of the effect of meteorological and environmental factors on the prevalence of wheezing diseases, which could be useful and crucial in predicting the pattern of children wheezing diseases. Diverse seasons or regions may show different correlations between wheezing diseases and certain meteorological and environmental factors. Meteorological and environmental factors had a crucial influence on the prevalence of respiratory viruses such as RSV, HRV, and hMPV^28–30^, which were the main pathogens of wheezing diseases in children^13–16^. As there was an association among meteorological and environmental factors, viruses, and wheezing diseases, we included wheezing children with virus infection. The ARIMA model used in this study predicted the prevalence of wheezing diseases on the basis of temperature changes, which could be used as a reference for predicting the incidence of wheezing diseases and activity of virus in similar climatic and environmental conditions. The outcome was based on several types of diseases which were mainly caused by viral infections and inextricably linked; however, other potential factors such as characteristics of different diseases and age should also be considered. Although there were many studies focused on the relationship between daily meteorological factors and wheezing diseases, it is not practical to use daily climate to predict disease development clinically, and the climate in Suzhou changes little in the same month. Therefore, we predicted and evaluated the number of wheezing children by month at present. We also analyzed the effects of seasonal variation and wheezing to better understand the impact of meteorological and environmental factors on asthmatic disease cases, and the results of seasonal variation was consistent with that of month. This model further validated the results of this study on the correlation between temperature and wheezing in children. Therefore, it is of great significance to integrate meteorological and environmental research into public health thinking and prevention.

## Conclusion

This study described the monthly and seasonal patterns of children wheezing disease and suggested that meteorological and environmental factors, particularly temperature, were associated with virus-induced wheezing diseases in children. On the basis of this, we further estimated the effect of temperature on wheezing diseases using ARIMA models. It could be effectively used as an early warning indicator for the occurrence of children wheezing diseases and for clinical application.

## Supplementary Information

Supplementary Table S1.

Supplementary Table S2.

Supplementary Table S3.

Supplementary Table S4.

Supplementary Table S5.

Supplementary Table S6.

Supplementary Table S7.

## Data Availability

The datasets used and/or analyzed during the current study are available from the corresponding author on reasonable request.

## Abbreviations

ADV: Adenovirus
ARIMA: Autoregressive integrated moving average models
CO: Carbon monoxide
HBoV: Human bocavirus
HMPV: Human metapneumovirus
HRV: Human rhinovirus
Inf: Influenza virus
MP: Mycoplasma pneumoniae
NO_2_: Nitrogen dioxide
O_3_: Ozone
Pinf: Parainfluenza virus
PM: Particulate matter
RSV: Respiratory syncytial virus
SO_2_: Sulfur dioxide
VIF: Variance inflation factor
