# Supplementary material for: Association of children wheezing diseases with meteorological and environmental factors in Suzhou, China
Source: Sci Rep. 2022 Mar 23;12:5018. doi: 10.1038/s41598-022-08985-5 (PMC8943037; doi:10.1038/s41598-022-08985-5)
Supplement: Supplementary file 1 — Supplementary Table S1. [file 41598_2022_8985_MOESM1_ESM.docx]

**Supplementary Table S1.** Seasonality of Wheezing children during from 2013 to 2017(‾x ±s)

| **Season** | **Wheezing children(n)** |
| --- | --- |
| Spring | 165 ± 27 |
| Summer | 115 ± 22 |
| Autumn | 165 ± 40 |
| Winter | 204 ± 60 |
| F value | 4.170 |
| P value | 0.023 |
