# Supplementary material for: Association of children wheezing diseases with meteorological and environmental factors in Suzhou, China
Source: Sci Rep. 2022 Mar 23;12:5018. doi: 10.1038/s41598-022-08985-5 (PMC8943037; doi:10.1038/s41598-022-08985-5)
Supplement: Supplementary file 2 — Supplementary Table S2. [file 41598_2022_8985_MOESM2_ESM.docx]

**Supplementary Table S2.** Correlation between seasonal meteorological factors and wheezing diseases in children

| **Meteorological parameter** | **Standardized Coefficients** | **t** | **Sig** | **VIF** |
| --- | --- | --- | --- | --- |
| Female ratio | 0.044 | 0.212 | 0.835 | 1.209 |
| Average seasonal age(months) | -0.149 | -0.736 | 0.474 | 1.159 |
| Mean temperature | -0.775 | -3.503 | 0.004 | 1.395 |
| Relative humidity | 0.211 | 0.998 | 0.335 | 1.268 |
| Wind velocity | 0.169 | 0.841 | 0.414 | 1.151 |

Dependent variable: number of wheezing diseases, t: t value, sig: significance,

VIF: variance inflation factor
