# Supplementary material for: Association of children wheezing diseases with meteorological and environmental factors in Suzhou, China
Source: Sci Rep. 2022 Mar 23;12:5018. doi: 10.1038/s41598-022-08985-5 (PMC8943037; doi:10.1038/s41598-022-08985-5)
Supplement: Supplementary file 3 — Supplementary Table S3. [file 41598_2022_8985_MOESM3_ESM.docx]

**Supplementary Table S3.** ARIMA models for the number of wheezing children

| **Parameter** | **Lag time** | **Estimate** | **S.E.** | **P-value** | **Stationary**  **R^2^ value** | **Normalized BIC** |
| --- | --- | --- | --- | --- | --- | --- |
| Temperature | Lag 0 | −0.037 | 0.007 | <0.001 | 0.556 | 5.575 |
|  | Lag 1 | -1.240 | 0.331 | 0.001 | 0.238 | 6.007 |
|  | Lag 2 | -0.487 | 0.372 | 0.196 | 0.038 | 6.234 |
| Rainfall | Lag 0 | -0.089 | 0.033 | 0.01 | 0.037 | 6.228 |
|  | Lag 1 | -0.071 | 0.032 | 0.033 | 0.097 | 6.177 |
|  | Lag 2 | -0.054 | 0.033 | 0.108 | 0.058 | 6.213 |
| PM_2.5_ | Lag 0 | 0.427 | 0.146 | 0.005 | 0.057 | 6.205 |
|  | Lag 1 | 0.428 | 0.138 | 0.003 | 0.177 | 6.085 |
|  | Lag 2 | 0.278 | 0.146 | 0.063 | 0.076 | 6.193 |
| PM_10_ | Lag 0 | 0.332 | 0.113 | 0.005 | 0.158 | 6.204 |
|  | Lag 1 | 0.297 | 0.109 | 0.009 | 0.142 | 6.126 |
|  | Lag 2 | 0.207 | 0.113 | 0.073 | 0.071 | 6.199 |
| NO_2_ | Lag 0 | 1.120 | 0.253 | <0.001 | 0.299 | 6.020 |
|  | Lag 1 | 0.977 | 0.251 | <0.001 | 0.252 | 5.989 |
|  | Lag 2 | 0.701 | 0.270 | 0.013 | 0.133 | 6.130 |
| CO | Lag 0 | 52.713 | 15.937 | 0.002 | 0.192 | 6.162 |
|  | Lag 1 | 52.580 | 15.068 | 0.001 | 0.213 | 6.040 |
|  | Lag 2 | 37.723 | 15.980 | 0.023 | 0.112 | 6.154 |
| O_3_ | Lag 0 | -0.411 | 0.090 | <0.001 | 0.313 | 6.001 |
|  | Lag 1 | -0.403 | 0.086 | <0.001 | 0.329 | 5.879 |
|  | Lag 2 | -0.282 | 0.097 | 0.006 | 0.160 | 6.099 |
