# Supplementary material for: Association of children wheezing diseases with meteorological and environmental factors in Suzhou, China
Source: Sci Rep. 2022 Mar 23;12:5018. doi: 10.1038/s41598-022-08985-5 (PMC8943037; doi:10.1038/s41598-022-08985-5)
Supplement: Supplementary file 4 — Supplementary Table S4. [file 41598_2022_8985_MOESM4_ESM.docx]

**Supplementary Table S4.** Monthly average of Wheezing children from 2013 to 2017(‾x ±s)

| **Month** | **Wheezing children(n)** |
| --- | --- |
| January | 77 ± 42 |
| February | 65 ± 18 |
| March | 60 ± 17 |
| April | 58 ± 11 |
| May | 48 ± 12 |
| June | 43 ± 10 |
| July | 36 ± 10 |
| August | 37 ± 6 |
| September | 44 ± 18 |
| October | 59 ± 15 |
| November | 62 ± 13 |
| December | 63 ± 28 |
| F value | 2.186 |
| P value | 0.031 |
