# Supplementary material for: Association of children wheezing diseases with meteorological and environmental factors in Suzhou, China
Source: Sci Rep. 2022 Mar 23;12:5018. doi: 10.1038/s41598-022-08985-5 (PMC8943037; doi:10.1038/s41598-022-08985-5)
Supplement: Supplementary file 5 — Supplementary Table S5. [file 41598_2022_8985_MOESM5_ESM.docx]

**Supplementary Table S5.** Associations between seasonal meteorological factors and wheezing diseases in children (Pearson correlation)

|  | **Mean temperature**  **(℃)** | **Relative humidity**  **(%)** | **Total rainfall**  **(mm)** | **Total sunshine**  **(h)** | **Wind velocity**  **(m/s)** |
| --- | --- | --- | --- | --- | --- |
| Wheezing children (n) | -0.661** | -0.137 | -0.598** | -0.270 | -0.014 |

*P<0.05

**P<0.01
