# Supplementary material for: Association of children wheezing diseases with meteorological and environmental factors in Suzhou, China
Source: Sci Rep. 2022 Mar 23;12:5018. doi: 10.1038/s41598-022-08985-5 (PMC8943037; doi:10.1038/s41598-022-08985-5)
Supplement: Supplementary file 6 — Supplementary Table S6. [file 41598_2022_8985_MOESM6_ESM.docx]

**Supplementary Table S6.** Associations between seasonal environmental factors and wheezing diseases in children (Pearson correlation)

|  | **PM_2.5_**  **(µg/m^3^)** | **PM_10_**  **(µg/m^3^)** | **NO_2_**  **(µg/m^3^)** | **SO_2_**  **(µg/m^3^)** | **CO**  **(µg/m^3^)** | **O_3_**  **(µg/m^3^)** |
| --- | --- | --- | --- | --- | --- | --- |
| Wheezing children(n) | 0.530* | 0.587** | 0.600** | 0.365 | 0.546* | -0.436 |

*P<0.05

**P<0.01
