# Supplementary material for: Association of children wheezing diseases with meteorological and environmental factors in Suzhou, China
Source: Sci Rep. 2022 Mar 23;12:5018. doi: 10.1038/s41598-022-08985-5 (PMC8943037; doi:10.1038/s41598-022-08985-5)
Supplement: Supplementary file 7 — Supplementary Table S7. [file 41598_2022_8985_MOESM7_ESM.docx]

**Supplementary Table S7.** Correlation between seasonal environmental factors and wheezing diseases in children

| **Environmental parameter** | **Standardized Coefficients** | **T** | **Sig** | **VIF** |
| --- | --- | --- | --- | --- |
| Female ratio | 0.097 | 0.366 | 0.720 | 1.621 |
| Average seasonal age(months) | -0.248 | -1.004 | 0.334 | 1.393 |
| NO_2_ | 0.489 | 1.203 | 0.250 | 3.766 |
| SO_2_ | 0.027 | 0.084 | 0.934 | 2.301 |
| CO | 0.140 | 0.321 | 0.753 | 4.346 |
| O_3_ | -0.023 | -0.058 | 0.955 | 3.500 |

Dependent variable: number of wheezing diseases, t: t value, sig: significance,

VIF: variance inflation factor
